# Withaferin A Attenuates Muscle Cachexia Induced by Angiotensin II Through Regulating Pathways Activated by Angiotensin II

**DOI:** 10.3390/cells14040244

**Published:** 2025-02-08

**Authors:** Sham S. Kakar, Vasa Vemuri, Mariusz Z. Ratajczak

**Affiliations:** 1 Department of Physiology, University of Louisville, Louisville, KY 40202, USA; sreevasa.vemuri@louisville.edu; 2 Brown Cancer Center, University of Louisville, Louisville, KY 40202, USA; mariusz.ratajczak@louisville.edu; 3 Department of Medicine, University of Louisville, Louisville, KY 40202, USA

**Keywords:** muscle cachexia, Ang II, cytokines, muscle function, withaferin A

## Abstract

Cachexia is a multifactorial syndrome characterized by severe muscle wasting and is a debilitating condition frequently associated with cancer. Previous studies from our group revealed that withaferin A (WFA), a steroidal lactone, mitigated muscle cachexia induced by ovarian tumors in NSG mice. However, it remains unclear whether WFA’s protective effects are direct or secondary to its antitumor properties. We developed a cachectic model through continuous angiotensin II (Ang II) infusion in C57BL/6 mice to address this issue. Ang II infusion resulted in profound muscle atrophy, evidenced by significant reductions in grip strength and in the TA, GA, and GF muscle mass. Molecular analyses indicated elevated expression of inflammatory cytokines (TNFα, IL-6, MIP-2, IL-18, IL-1β), NLRP3 inflammasome, and genes associated with the UPS (MuRF1, MAFBx) and autophagy pathways (Bacl1, LC3B), along with suppression of anti-inflammatory heme oxygenase-1 (HO-1) and myogenic regulators (Pax7, Myod1). Strikingly, WFA treatment reversed these pathological changes, restoring muscle mass, strength, and molecular markers to near-normal levels. These findings demonstrate that WFA exerts direct anti-cachectic effects by targeting key inflammatory and atrophic pathways in skeletal muscle, highlighting its potential as a novel therapeutic agent for cachexia management.

## 1. Introduction

Cachexia, a complication of inflammatory diseases, is marked by a significant depletion of skeletal muscle mass and, to a lesser extent, adipose tissue [1,2]. In oncology, cachexia impacts up to 80% of cancer patients and accounts for nearly 30% of cancer-related deaths [1,2]. This syndrome is closely linked to poor clinical outcomes, decreased quality of life, and limited tolerance to antineoplastic therapies [1,2,3]. Key clinical manifestations include loss of appetite, anemia, severe weakness, and disturbances in lipid and protein metabolism [1,4,5]. Recent studies have also emphasized the pathological remodeling of various organs, including the heart, as a defining feature of cancer cachexia. Despite progress in understanding the molecular pathways associated with cachexia, a unifying mechanism remains elusive, and no approved therapeutic interventions are currently available [6].

Withaferin A (WFA), a steroidal lactone derived from *Withania somnifera* (commonly known as Ashwagandha or winter cherry), is recognized for its potent anti-inflammatory and cardioprotective properties [7,8,9]. WFA has effectively inhibited cancer cell proliferation, invasion, and metastasis across various oncological models [10,11,12,13,14]. Although previous research, including studies from our group and others [15,16,17,18,19,20], has extensively explored the pharmacological effects of WFA, its potential application in alleviating cancer-induced cachexia or cardiac cachexia has yet to be investigated.

Our earlier work demonstrated the therapeutic potential of WFA in addressing ovarian cancer-induced skeletal muscle cachexia in mice [21,22]. Our results revealed that cancer-bearing mice, generated by the peritoneal injection of metastatic A2780 ovarian cancer cells, exhibited a substantial enhancement in survival rates, a restoration of grip strength, and an improvement in myofibrillar cross-sectional area upon treatment with WFA. Furthermore, WFA prevented the slow-to-fast myofiber type conversion typically associated with cancer cachexia and reduced NF-κB-mediated pro-inflammatory cytokines and NLRP3 inflammasome signaling [21]. These outcomes collectively illustrate the potential therapeutic role of WFA in alleviating the devastating effects of cancer-induced cachexia, with implications for the broader management of cancer-associated wasting syndromes.

To investigate whether WFA’s protective effects on muscle are directly linked to its actions on skeletal muscle or are secondary to its antitumor properties, we employed a pharmacological angiotensin II (Ang II) infusion model of cachexia independent of tumor burden [23]. Ang II, a crucial mediator of the renin-angiotensin system, is elevated in chronic diseases such as cancer, chronic kidney disease, and heart failure [24,25,26,27]. In tumor-bearing animals, increased circulating Ang II levels correlate with exacerbated cachexia severity [22]. Continuous Ang II infusion has been shown to drive skeletal muscle wasting and worsen cachexia [24,28,29]. In this study, we developed a cachexia model using osmotic minipumps for sustained systemic Ang II infusion, allowing us to assess the direct anti-cachectic effects of WFA on skeletal muscle.

## 2. Materials and Methods

### 2.1. Ethical Approval

All animal experiments conducted in this study adhered strictly to the National Institutes of Health guidelines for the ethical treatment and handling of laboratory animals. The Institutional Animal Care and Use Committee (IACUC) of the University of Louisville pre-approved the experimental protocols under protocol #19653.

### 2.2. Implantation of Osmotic Pumps in C57BL/6J Mice

Eleven-week-old female C57BL/6J mice were procured from Jackson Laboratory and housed in the animal facility at the University of Louisville. Mice were randomly stratified into two groups (n = 10). Baseline body weight measurements and forelimb and total grip strength were conducted using a digital grip strength meter, as previously described [21].

Osmotic minipumps (ALZET Model 1004 Osmotic Minipumps, Cupertino, CA, USA) were implanted subcutaneously in the suprascapular region to ensure consistent delivery of angiotensin II (Ang II; 1000 ng/kg/min) or an equivalent volume of sterile saline. Minipumps were calibrated to release their contents at a constant rate of 0.1 μL/hour for four weeks [23]. Following a one-week post-implantation period, mice were further subdivided (n = 5 per group) and administered either withaferin A (WFA; 4 mg/kg in 100 μL, intraperitoneally) or vehicle (10% DMSO, 90% glycerol trioctanoate) once every three days for the remaining three weeks of the study, as previously detailed [29].

### 2.3. Grip Strength Measurement

Grip strength was assessed weekly to monitor changes in functional muscle performance as described previously [21]. Before each measurement, mice were weighed using a digital scale, and forelimb and total limb grip strength were normalized to body weight. The grip strength measurement was performed using a digital grip strength meter (Columbus Instruments, Columbus, OH, USA). To assess forelimb grip strength, mice were allowed to grasp a forepaw assembly bar, and tension was applied by gently pulling the tail until the mice released the bar. The mice were allowed to hold the complete paw assembly for total limb grip strength before being similarly pulled. Each measurement consisted of five consecutive trials per mouse with a 30–40 s recovery period between trials. The peak grip strength from each trial was recorded, and the average of the five trials was calculated. Acclimatization of the mice to the testing apparatus occurred for two minutes before each session. Grip strength measurements provided insights into the effects of Ang II infusion and subsequent WFA treatment. The total and forelimb grip strengths were measured weekly, avoiding habituation to the grip strength analyses.

### 2.4. Tissue Collection and Histological Analysis

After four weeks of osmotic pump implantation, mice were euthanized, and tissues—including the tibialis anterior (TA), gastrocnemius (GA), quadratus femoris (QF), lungs, kidneys, and liver—were collected. Tissues were snap-frozen in liquid nitrogen and stored at −80 °C until analysis. Select tissues were embedded in OCT compound, sectioned to a thickness of 5 μm using a cryostat at −20 °C (Epredia Microm HM525), and mounted onto glass slides (two sections on each slide). Transverse sections from the mid-belly of the TA were stained with hematoxylin and eosin (H&E) for morphological analysis. Myofiber cross-sectional area (CSA) of the H&E-stained TA muscle sections was quantified using Fiji software (National Institutes of Health), analyzing approximately 500–700 myofibers per muscle, as described previously [21].

### 2.5. RNA Extraction and Quantitative Real-Time PCR (qRT-PCR)

Manufacturer’s instructions were used to extract total RNA from the GA muscle (due to limited TA tissue availability) using the RNeasy Fibrous Tissue Mini Kit (Qiagen; Cat #74704). Approximately 25–30 mg of GA tissue was homogenized using a polytron homogenizer in the extraction buffer provided with the kit. RNA was purified using the supplied column and quantified with a NanoDrop UV-Vis spectrophotometer (Fisher Scientific). First-strand cDNA was synthesized from 1 μg of total RNA using the iScript cDNA Synthesis Kit (Bio-Rad; Cat #170-8891). Gene expression levels were quantified using SYBR green dye and specific primers (Table 1) in a CFX-Connect Real-Time System (Bio-Rad). As described previously, beta-actin served as the internal reference gene for normalization [21].

### 2.6. Graphical Display and Statistical Analysis

Statistical analyses were performed using appropriate methods based on the experimental design. An unpaired two-tailed t-test with Welch’s correction was utilized to compare the two groups. For datasets involving three or more groups with a single experimental factor, one-way analysis of variance (ANOVA) followed by Tukey’s Honestly Significant Difference Test (HSDT) was applied. Two-way ANOVA with Tukey’s multiple comparisons test was conducted for experiments involving four or more groups with two experimental factors. Graphical representation and statistical computations were performed using GraphPad Prism 8.3.0 software for Mac (La Jolla, CA, USA), as detailed in prior studies [21].

## 3. Results

### 3.1. WFA Reverses the Muscle Cachexia Induced by Ang II

**Figure 1:**
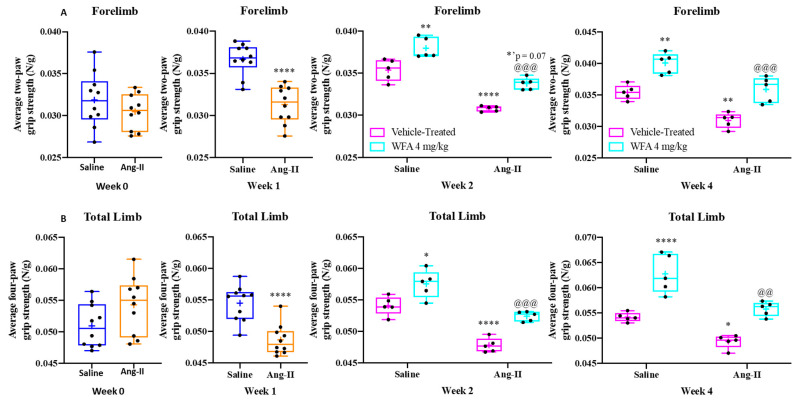
Withaferin A effectively restores grip strength impaired by Ang II. The normalized average grip strength, including (**A**) forelimb and (**B**) total limb strength, was assessed across Ang II-infused vehicle-treated groups and saline-infused controls, as well as the WFA-treated group (4 mg/kg) at baseline (week 0) and subsequent time points (week 1, week 2, and week 4) after osmotic pump implantation (N = 5 per group). Statistical analysis utilizing two-way ANOVA and Tukey’s multiple comparison tests revealed significant differences between groups. Statistical significance is indicated as follows: * *p* < 0.05, ** *p* < 0.01, **** *p* < 0.0001, comparing Ang II-infused vehicle-treated groups to saline-infused controls. Additionally, ^@@^ *p* < 0.01, ^@@@^ *p* < 0.001 denote significant differences between Ang II-infused WFA-treated groups and Ang II-infused vehicle-treated groups. N = 5/group.

Our previous investigations demonstrated that A2780 ovarian cancer cells induce skeletal muscle cachexia in NSG mice and that the administration of WFA effectively mitigates these pathological changes [21]. In this study, we utilized an Ang II infusion model to validate the induction of cachexia and evaluate the anti-cachectic potential of WFA. Prior findings showed no significant difference between WFA doses of 2 mg/kg and 4 mg/kg [21], so we focused exclusively on the 4 mg/kg dose in this study. Baseline measurements of forelimb and total grip strength revealed no significant differences among groups receiving saline or Ang II infusion. However, after one week of Ang II infusion via osmotic pumps, a marked reduction in both forelimb and total grip strength was observed in Ang II-infused animals compared to saline-infused controls. Notably, treatment with WFA (4 mg/kg), initiated one-week post-implantation of osmotic pumps, significantly restored forelimb and total grip strength in Ang II-infused animals. This improvement persisted throughout the experimental period (4 weeks), contrasting with the sustained decline in grip strength observed in untreated Ang II-infused animals. These findings suggest that WFA effectively attenuates Ang II-induced muscle weakness (Figure 1). Additionally, WFA treatment significantly enhanced forelimb and total grip strength in saline-infused animals compared to vehicle-treated controls, as determined by two-way ANOVA followed by Tukey’s multiple comparison test (Figure 1). This observation aligns with our prior studies using the cancer cachexia model, highlighting the consistent myogenic potential of WFA, potentially through the activation of satellite muscle cells [21].

**Figure 2:**
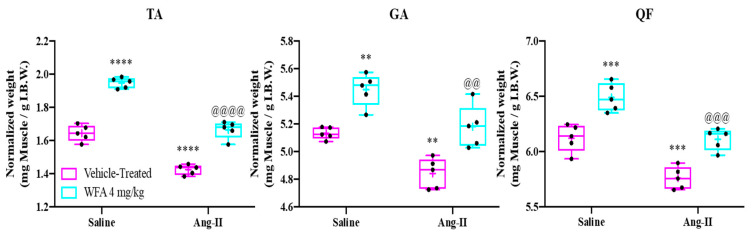
Withaferin A reduces muscle weight loss caused by Ang II infusion. The graph displays the wet weights of the tibialis anterior (TA), gastrocnemius (GA), and quadriceps femoris (QF) muscles, normalized to initial body weight (IBW) to account for baseline variability among experimental groups (N = 5 per group). Ang II infusion significantly decreased normalized muscle weights compared to the saline-infused vehicle-treated controls. Treatment with withaferin A (WFA) significantly recovered muscle mass in Ang II-infused animals and increased muscle weights in saline-infused animals. Statistical significance was assessed using two-way ANOVA followed by Tukey’s multiple comparison post hoc analysis. Symbols denote: ** *p* < 0.01; *** *p* < 0.001; **** *p* < 0.0001 for comparisons between Ang II-infused vehicle-treated and saline-infused groups, and ^@@^ *p* < 0.01; ^@@@^ *p* < 0.001; ^@@@@^ *p* < 0.0001 for comparisons between Ang II-infused WFA-treated and Ang II-infused vehicle-treated groups.

After the experimental period, selected lower extremity muscles, including the tibialis anterior (TA), gastrocnemius (GA), and quadriceps femoris (QF), were harvested and weighed to evaluate changes in muscle mass (Figure 2). To account for baseline differences in body size and muscle mass among individual mice, the muscle weights were normalized to initial body weight (IBW). A pronounced reduction in the normalized weights of the TA, GA, and QF muscles was observed in Ang II-infused animals compared to their saline-infused, vehicle-treated counterparts, indicating significant muscle wasting induced by Ang II infusion. Within the saline-infused cohort, treatment with WFA resulted in a marked increase in muscle mass compared to the saline vehicle-treated group, underscoring the anabolic potential of WFA even in non-cachectic conditions. Furthermore, the administration of WFA significantly mitigated muscle loss in the Ang II-infused groups, as evidenced by the substantial increase in the normalized wet weights of the TA, GA, and QF muscles compared to the Ang II-infused, vehicle-treated group. These findings highlight the efficacy of WFA in preserving and restoring muscle mass in the context of Ang II-induced cachexia (Figure 2).

**Figure 3:**
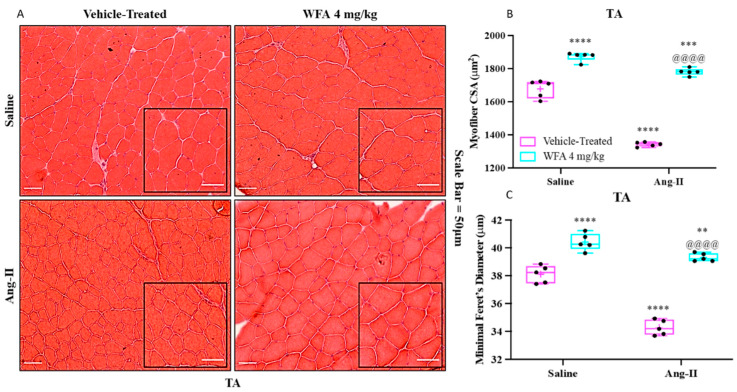
Withaferin A restores myofibrillar integrity in skeletal muscle. (**A**) Representative transverse sections of the tibialis anterior (TA) muscle, stained with hematoxylin and eosin (H&E), highlight structural differences among the treatment groups. The images illustrate how withaferin A affects muscle architecture, showing visible differences in myofiber size and organization. Insets provide magnified views of selected areas from the larger images to effectively showcase the cellular and tissue-level changes. Scale bar = 50 μm. (**B**) Quantitative analysis of myofiber cross-sectional area (CSA) clarifies the impact of withaferin A treatment on myofiber size. (**C**) Minimal Feret’s diameter measurements further quantify myofiber integrity, assessing the structural alterations in the TA muscle. Together, these parameters reveal the restorative effects of withaferin A on muscle structure and integrity. Data represent a sample size of N = 5 per group. Statistical analysis indicates significance at ** *p* < 0.01, *** *p* < 0.001, and **** *p* < 0.0001, marking significant differences between Ang II-infused groups and their saline-infused counterparts. Additionally, ^@@@@^ *p* < 0.0001 highlights a significant difference between Ang II-infused, WFA-treated groups and Ang II-infused vehicle-treated groups. Statistical assessments were conducted using two-way ANOVA followed by Tukey’s multiple comparison test for post hoc analysis.

To corroborate the observed changes in muscle strength and weight following WFA treatment, histological analysis of myofibril size in the tibialis anterior (TA) muscle was conducted using hematoxylin and eosin (H&E) staining (Figure 3A). Consistent with the improvements in grip strength, WFA treatment in saline-infused animals significantly increased the average myofibril cross-sectional area (CSA) of the TA muscle compared to the saline-infused vehicle-treated group (Figure 3B). Conversely, the average CSA of the TA muscle in the Ang II-infused vehicle-treated group was markedly reduced relative to all saline-infused groups, both vehicle- and WFA-treated. Notably, WFA treatment in the Ang II-infused group fully restored the myofibril CSA to levels comparable to the saline-infused controls (Figure 3B). To enhance the reliability of these findings, the minimal Feret’s diameter, a complementary measure of myofiber size, was also assessed (Figure 3C). The trends and statistical significance observed in the minimal Feret’s diameter closely mirrored those of the CSA, confirming the accuracy and consistency of the histological evaluation (Figure 3C). Although the observed restoration of myofiber size by WFA aligns with improvements in muscle mass and functional strength, it is essential to note that a direct correlation between muscle size and strength remains a debated topic in the current literature [30,31]. Nonetheless, the reduction in myofiber CSA observed in Ang II-infused animals corresponds to the decline in muscle weight and grip strength, further supporting the potential of WFA to mitigate Ang II-induced muscle atrophy.

### 3.2. WFA Reverses the Increase in Inflammatory Cytokines by Ang II

Cytokines are pivotal in regulating intercellular communication, functioning locally and systemically [26]. During conditions such as cancer and infection, numerous cytokines are upregulated in circulation and within local tissues. These inflammatory mediators drive muscle atrophy and activate transcription factors that propagate catabolic processes [6,32,33,34]. Specifically, cytokines like TNF-α, IL-6, IL-18, IL-1β, and INF-γ are implicated in cancer cachexia, promoting elevated energy expenditure, reduced appetite, and skeletal muscle degradation [35].

Our previous studies demonstrated a significant increase in TNF-α, IL-6, IL-18, IL-1β, and INF-γ in the skeletal muscle of tumor-bearing animals, which normalized after treatment with withaferin A (WFA) [21]. Ang II is a recognized inflammatory mediator known to elevate circulating levels of pro-inflammatory cytokines in both skeletal and cardiac muscle [27]. To investigate the inflammatory response triggered by Ang II infusion and the modulatory effects of WFA, we examined the expression of key cytokine genes in the gastrocnemius (GA) muscle.

**Figure 4:**
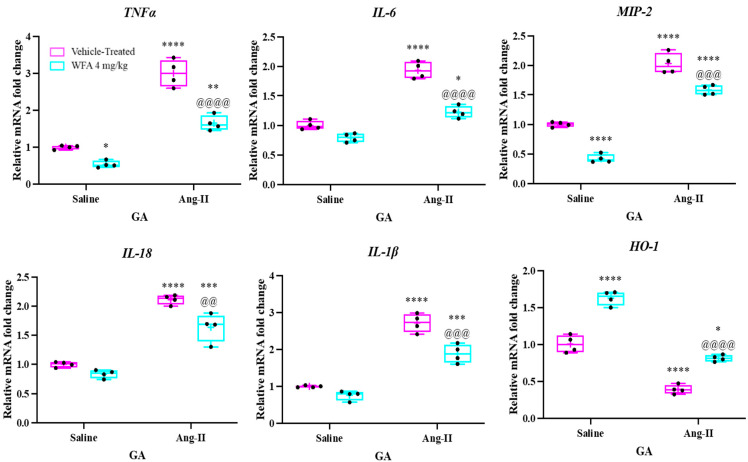
Withaferin A influences the expression of inflammatory cytokines in gastrocnemius (GA) muscles induced by Ang II. Relative mRNA expression levels of pro-inflammatory cytokines (TNF-α, IL-6, MIP-2, IL-18, IL-1β) and the anti-inflammatory cytokine HO-1 in GA muscles were measured. Data are presented as the mean ± SD, with individual data points shown as black circles (N = 5 per group). Statistical significance was assessed using two-way ANOVA followed by Tukey’s multiple comparison test. * *p* < 0.05; ** *p* < 0.01; *** *p* < 0.001; **** *p* < 0.0001 indicate significant differences between the Ang II-infused and saline-infused vehicle-treated groups. ^@@^ *p* < 0.01, ^@@@^ *p* < 0.001, ^@@@@^ *p* < 0.0001 denote significant differences between the Ang II-infused WFA-treated group and the Ang II-infused vehicle-treated group.

As shown in Figure 4, relative mRNA expression levels of TNF-α, IL-6, MIP-2 (the mouse version of human IL-8), IL-18, and IL-1β were significantly increased in the GA muscles of Ang II-infused mice compared to those receiving saline infusions. Importantly, WFA treatment significantly reduced the expression of these pro-inflammatory cytokines, bringing their levels back to baseline. In contrast, the anti-inflammatory cytokine heme oxygenase-1 (HO-1) was downregulated in the GA muscles of Ang II-infused mice but returned to control levels after WFA treatment.

The observed increase in inflammatory cytokine expression in Ang II-infused skeletal muscles aligns with findings from other studies [27,36]. Most genes analyzed (Figure 4) showed significant upregulation in Ang II-infused animals compared to saline-infused controls, regardless of treatment with vehicle or WFA. Importantly, WFA effectively normalized HO-1 expression. These results suggest that the upregulation of inflammatory cytokines in skeletal muscle may contribute to Ang II-induced muscle cachexia. WFA alleviates these effects by reducing inflammatory cytokine levels and restoring anti-inflammatory mechanisms.

### 3.3. WFA Targets NLRP3 Inflammasomes in Reversing Muscle Cachexia

**Figure 5:**
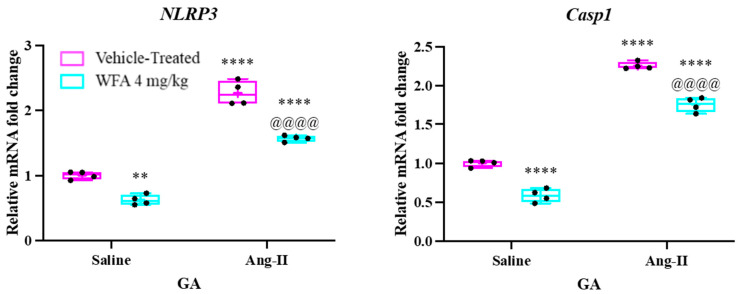
Withaferin A reverses Ang II-induced activation of the NLRP3 inflammasome. Mice were infused with Ang II, as outlined in the Materials and Methods section. After 4 weeks of continuous Ang II infusion, gastrocnemius muscle tissues were collected from each of the four experimental groups. The mRNA expression levels of NLRP3 and Caspase-1 were quantified using quantitative reverse transcription polymerase chain reaction (qRT-PCR). Data are presented as mean ± SD (n = 5 per group). Statistical significance was assessed by comparing the Ang II-infused groups to the saline-infused controls, with ** *p* < 0.01 and **** *p* < 0.0001. Additionally, ^@@@@^ *p* < 0.0001 denotes a significant difference between the Ang II-infused vehicle-treated and Ang II-infused WFA-treated groups.

There is increasing evidence supporting the role of inflammasomes in the pathophysiology of various inflammatory disorders. These complexes are activated through a series of protein oligomerization events that recognize specific molecular patterns resulting from cellular damage, infections, or disruptions in homeostasis. The NOD-like receptor family of proteins (NLRPs), which includes NLRP1, NLRP3, NLRC4, and absent in melanoma 2 (AIM2), are the main components of inflammasomes, with NLRP3 being the most extensively studied [37]. A crucial step in inflammasome activation is the upregulation of NLRP3 expression [38]. The activation of the NLRP3 inflammasome requires two signals: one initiated by nuclear factor kappa B (NFκB) signaling and the second by factors such as mitochondrial reactive oxygen species [39]. Once activated, the NLRP3 inflammasome triggers the protease caspase-1 (CASP1), which processes and releases mature caspase-1 (CASP1 p20) and facilitates the production of proinflammatory cytokines, notably IL-18 and IL-1β [40]. Previous studies have shown that Ang II infusion in C57BL6 mice increases the levels of inflammatory cytokines and NLRP3 inflammasomes [34]. Our earlier work demonstrated that ovarian tumor generation in NSG mice led to elevated NLRP3 inflammasome levels in skeletal muscle, which were subsequently reduced with WFA treatment [21]. To explore the potential of WFA in modulating NLRP3 inflammasome activation in response to continuous Ang II infusion, we measured the expression of key NLRP3 inflammasome-associated proteins, including NLRP3 and Caspase-1 in gastrocnemius (GA) muscle tissues. As shown in Figure 5, NLRP3 and Caspase-1 mRNA expression was significantly elevated in the Ang II-infused group compared to the saline-infused group. However, WFA treatment in Ang II-infused mice significantly reduced the levels of NLRP3 and Caspase-1. These findings suggest that Ang II infusion activates the NLRP3 inflammasome, contributing to the development of muscle cachexia, and that WFA effectively inhibits this activation.

### 3.4. WFA Targets Several Signaling Pathways Leading to the Development of Muscle Cachexia

**Figure 6:**
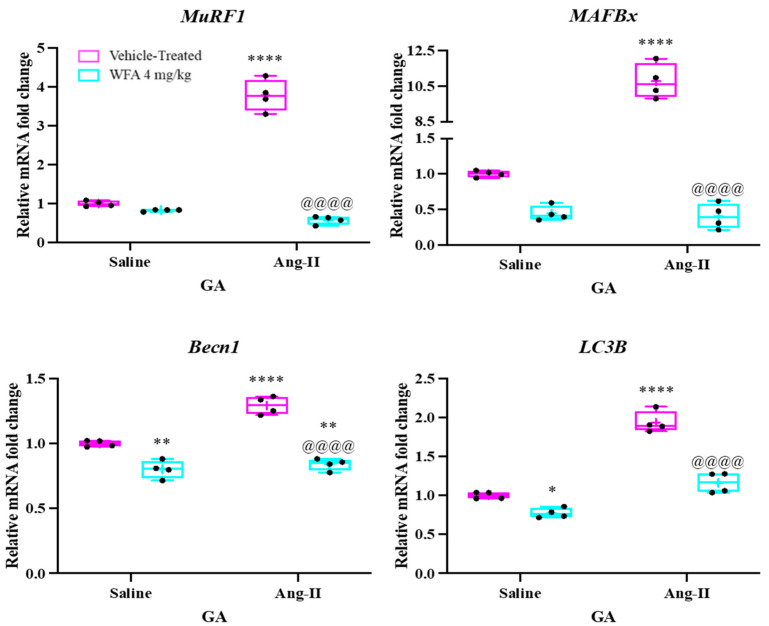
Withaferin A downregulates the activation of the ubiquitin–proteasome system (UPS) and autophagy-related genes. This figure displays relative mRNA levels of key markers linked to the UPS and autophagy in gastrocnemius (GA) muscles from both saline-infused and Ang II-infused groups. Data are presented as mean ± SD, with N = 5 per group. Statistical significance was assessed using two-way ANOVA followed by Tukey’s multiple comparison post hoc analysis. * *p* < 0.05, ** *p* < 0.01, **** *p* < 0.0001 indicate significant differences between Ang II-infused and saline-infused vehicle-treated groups. Additionally, ^@@@@^ *p* < 0.0001 shows significant differences from the corresponding values of the Ang II-infused WFA-treated group compared to the Ang II-infused vehicle-treated group.

Muscle RING-finger protein-1 (MuRF-1) and muscle atrophy F-box (MAFbx, also known as Atrogin-1) are crucial in driving muscle cell damage and atrophy. Both Atrogin-1 and MuRF-1 (Trim63) are E3 ubiquitin ligases, primarily expressed in skeletal and cardiac muscle tissues, and play essential roles in muscle remodeling processes. The transcription of Atrogin-1 and MuRF-1 genes is significantly upregulated during skeletal muscle atrophy, whereas reduced expression has been linked with cardiac hypertrophy. For example, Kadoguchi et al. [41] demonstrated that Ang II infusion into C57BL6 mice using osmotic minipumps significantly decreased body weight, hindlimb skeletal muscle mass, and muscle fiber cross-sectional area after 4 weeks. Their study also revealed considerable increases in MuRF-1 and Atrogin-1 expression in the skeletal muscles of Ang II-treated mice. Our previous studies further confirmed that the development of ovarian tumors in NSG mice led to a marked upregulation of MuRF1 and MAFbx in skeletal muscle. To explore whether withaferin A (WFA) can reduce the expression of these atrophy-related genes, we assessed the mRNA levels of MuRF1 and MAFbx in the gastrocnemius (GA) muscles of Ang II-infused animals. Our results indicate that the mRNA levels of both MuRF1 and MAFbx were significantly elevated in the GA muscles of Ang II-infused animals treated with vehicle, compared to saline-infused controls receiving either vehicle or WFA (Figure 6). Importantly, WFA treatment in Ang II-infused subjects significantly decreased the mRNA expression of both MuRF1 and MAFbx, returning levels to those of controls. These findings suggest that WFA effectively targets key atrophic genes, contributing to the reversal of muscle cachexia induced by Ang II (Figure 6).

In addition to the ubiquitin–proteasome system (UPS), autophagy is another critical process that contributes to skeletal muscle atrophy. During autophagy, the conversion of LC3B-I to LC3B-II facilitates its interaction with autophagic vesicles, playing a pivotal role in the degradation of cellular components [42]. The protein p62 serves as a substrate in autophagy by recognizing and binding to ubiquitinated proteins [43]. As autophagy is activated, p62 protein levels decrease, prompting transcriptional upregulation to restore the depleted protein pool. Beclin1, another key protein involved in autophagy, is essential for initiating autophagosome formation [44]. Studies by Augusto et al. [45] demonstrated a significant increase in LC3B-II levels in muscle from Ang II-infused mice compared to controls, while Yadav et al. [46] reported elevated levels of both Beclin1 and LC3B-II in response to Ang II, further supporting their involvement in the autophagic process [47]. In our study, qRT-PCR analysis revealed a significant increase in the expression levels of both Beclin1 and LC3B-II in the gastrocnemius (GA) muscles of Ang II-infused animals treated with vehicle, compared to saline-infused controls. However, treatment with withaferin A (WFA) significantly reduced the expression of both Beclin1 and LC3B-II, suggesting that these proteins contribute to muscle cachexia induced by Ang II and that WFA may inhibit their activation (Figure 6).

**Figure 7:**
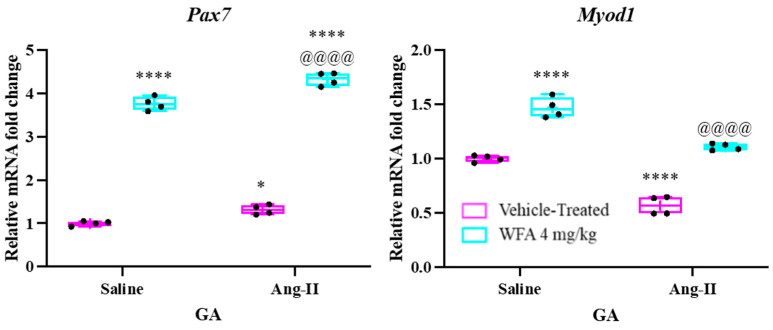
Withaferin A (WFA) reduces the expression of satellite cell-related genes in response to Ang II infusion. mRNA levels of Pax7 and Myod1 were measured in gastrocnemius (GA) muscles from saline-infused and Ang II-infused animals, both with and without WFA treatment. N = 5 per group. Data are presented as mean ± SD. * *p* < 0.05 and **** *p* < 0.0001 indicate significant differences from the corresponding value of the Ang II-infused vehicle-treated group, as determined by two-way ANOVA followed by Tukey’s multiple comparison test. ^@@@@^ *p* < 0.0001 indicates a significant difference comparing the Ang II-infused vehicle-treated group to the Ang II-infused WFA-treated group.

Skeletal muscle displays a remarkable ability to regenerate, primarily driven by muscle satellite cells that reside between the basal lamina and the sarcolemma of myofibers [48]. Upon muscle injury, these satellite cells are activated from their quiescent state and start proliferating. The proliferating satellite cells, also known as myogenic precursor cells (myoblasts), exit proliferation and differentiate into myocytes, fusing either with each other or with existing myofibers to repair the damaged muscle tissue [49]. The expression of key myogenic transcription factors, Pax7 and MyoD, serves as a marker for satellite cell differentiation. Pax7 + MyoD− cells are quiescent, Pax7 + MyoD+ cells are proliferating, and Pax7−MyoD+ cells undergo differentiation [50,51]. Our previous studies utilizing the NSG mouse tumor model demonstrated a significant increase in Pax7 expression and a notable decrease in Myod1 in the GA muscles of tumor-bearing, vehicle-treated mice compared to tumor-free controls [21]. In both tumor-free and tumor-bearing mice treated with WFA, we observed a significant increase in Pax7 levels and a notable rise in Myod1 expression compared to the vehicle-treated groups. This indicates that WFA may stimulate satellite cell differentiation through a yet-to-be-defined mechanism. In the present study, we found that Ang II infusion significantly decreased the mRNA levels of both Myod1 and Pax7 in GA muscles. However, treatment with WFA in Ang II-infused animals resulted in a significant increase in the expression of both genes. Interestingly, WFA alone, in saline-infused animals, also significantly elevated Pax7 and Myod1 mRNA levels. These findings suggest that WFA alleviates the effects of Ang II-induced muscle atrophy and enhances muscle repair and regeneration by promoting myogenesis (Figure 7).

## 4. Discussion

This study offers a thorough examination of the effects of withaferin A (WFA) on muscle atrophy caused by angiotensin II (Ang II), emphasizing its molecular mechanisms in skeletal muscle. Muscle atrophy is a complex process driven by intricate molecular signaling pathways, with both the ubiquitin–proteasome system (UPS) and autophagy recognized as key regulators [33]. Ang II infusion has been shown to induce muscle cachexia, noted for increased inflammatory cytokines, activation of the NLRP3 inflammasome, and upregulation of atrophic gene expression [26,28]. This study illustrates that WFA, a well-known anti-inflammatory and anti-catabolic agent, mitigates these pathological processes, indicating its potential as a therapeutic target for muscle wasting conditions [21,22].

Our results demonstrate that Ang II infusion significantly increased the expression of pro-inflammatory cytokines, including TNF-α, IL-6, IL-18, IL-1β, and IFN-γ in skeletal muscle, contributing to heightened muscle wasting. Treatment with WFA decreased the expression of these cytokines, supporting our hypothesis that WFA can modulate the inflammatory response and prevent the cascade of events leading to muscle degeneration [21,22]. Furthermore, we noted a significant reduction in the expression levels of NLRP3 inflammasome components, such as NLRP3 and Caspase-1, which are crucial mediators of inflammatory signaling [37,39]. These findings indicate that WFA may exert its protective effects by inhibiting the NLRP3 inflammasome pathway, a key regulator of muscle atrophy in response to Ang II.

Furthermore, the upregulation of the atrophic markers MuRF1 and MAFbx in skeletal muscle was significantly decreased by WFA treatment. These E3 ubiquitin ligases are crucial players in muscle protein degradation and are known to be elevated in response to systemic inflammation and muscle injury [28,33]. By targeting these atrophic genes, WFA seems to counteract the muscle wasting induced by Ang II, providing evidence of its potential to reverse or mitigate cachexia-related muscle loss. Our study also emphasizes the role of autophagy in Ang II-induced muscle atrophy, as indicated by increased expression of LC3B-II and Beclin-1 [42,44]. These autophagy-related proteins are upregulated in response to cellular stress, further supporting the notion that muscle atrophy involves both the UPS and autophagy pathways [45]. Importantly, WFA treatment significantly reduced the expression of these autophagic markers, suggesting that it may interfere with the autophagy-lysosomal pathway, thereby limiting the degradation of muscle proteins.

An additional layer of complexity in muscle regeneration and repair is the role of satellite cells, which are activated in response to injury or stress. The activation of satellite cells is crucial for muscle regeneration; however, its dysregulation can impair muscle repair and further exacerbate muscle atrophy. Our study demonstrated that Ang II infusion suppressed the expression of Pax7 and MyoD, two key transcription factors essential for satellite cell function. However, WFA treatment restored the levels of these markers and enhanced muscle repair, providing compelling evidence that WFA may promote myogenesis and muscle regeneration. The modulation of satellite cell activity in response to WFA treatment highlights the therapeutic potential of this compound in muscle wasting disorders.

This study highlights the potential of WFA as a therapeutic agent for muscle atrophy, but several limitations must be acknowledged. First, since the research was conducted in mice, further studies involving human populations are essential to validate the efficacy, safety, and optimal dosing of WFA. Additionally, while several pathways targeted by WFA have been identified, the precise molecular mechanisms underlying its effects still need clarification and require more investigation. The relatively small sample size of this study may restrict the generalizability of the findings, stressing the need for larger, multicenter trials to confirm these results. Finally, even though WFA shows promise in reducing muscle atrophy, its safety profile and potential side effects require comprehensive evaluation in future research.

## 5. Conclusions

Overall, this study identifies WFA as a potent modulator of various pathways involved in muscle atrophy, including inflammatory signaling, the UPS, autophagy, and satellite cell activation. Our findings provide compelling preclinical evidence supporting WFA’s potential as a novel therapeutic strategy for treating muscle-wasting conditions such as cancer cachexia and other forms of cachexia associated with chronic diseases. It is important to note that the current experiments were conducted in non-cancerous mice, and different results may be observed in cancer models, highlighting the need for further investigation. Future studies should aim to clarify the detailed molecular mechanisms through which WFA exerts its effects and evaluate its therapeutic potential in clinical settings, particularly regarding cancer-related muscle wasting. The ability of WFA to simultaneously target key pathways involved in muscle atrophy positions it as a promising candidate for development as a treatment for muscle-wasting disorders. This conclusion aligns with our initial hypothesis that WFA could provide a multifaceted approach to counteracting muscle atrophy, although further research is necessary to confirm its applicability across different disease contexts.

## Figures and Tables

**Table 1:** Gene-specific primer sequences.

Gene	Forward	Reverse
β-Actin	5′-CAGGCATTGCTGACAGGATG-3′	5′-TGCTGATCCACATCTGCTGG-3′
TNFα	5′-AGCACAGAAAGCATGATCCG-3′	5′-GCCACAAGCAGGAATGAGAA -3′
IL-6	5′-CCTTCTTGGGACTGATGCTGG-3′	5′-GCCTCCGACTTGTGAAGTGGT-3′
IL-1β	5′-TCACAGCAGCACATCAACAA-3′	5′-TGTCCTCATCCTGGAAGGTC-3′
MIP-2	5′-CCACTCTCAAGGGCGGTCAAA-3′	5′-TACGATCCAGGCTTCCCGGGT-3′
IL-18	5′-CGGCCTCTATTTGAAGATATGAC-3′	5′-CCATACCTCTAGGCTGGCTA-3′
HO-1	5′-ATGCCCCACTCTACTTCCCTGAGGAGC TG-3′	5′-TAGTGCTGTGTGGCTGGCGTGCAA-3′
NLRP3	5′-AGAAGAGACCACGGCAGAAG-3′	5′-CCTTGGACCAGGTTCAGTGT-3′
CasP1	5′-CACAGCTCTGGAGATGGTGA-3′	5′-GGTCCCACATATTCCCTCCT-3′
MuRF1	5′-TAACTGCATCTCCATGCTGGTG-3′	5′-TGGCGTAGAGGGTGTCAAACTT-3′
MAFbx (Atrogin I)	5′-AAGGCTGTTGGAGCTGATAGCA-3′	5′-CACCCACATGTTAATGTTGCCC-3′
BECLN1	5′-TGAAATCAATGCTGCCTGGG-3′	5′-CAGAACAGTATAACGGCAACTCC-3′
LC3B	5′-CTGGTGAATGGGCACAGCATG-3′	5′-CGTCCGCTGGTAACATCCCTT-3′
PAX7	5′-CAGTGTGCCATCTACCCATGCTTA-3′	5′-GGTGCTTGGTTCAAATTGAGCC-3′
Myod1	5′-TGGGATATGGAGCTTCTATCGC-3′	5′-GGTGAGTCGAAACACGGATCAT-3′

## Data Availability

Data contained within the article will be available on request.
